# Synergistic pathways for health investment and economic development in China: a fuzzy-set qualitative comparative analysis

**DOI:** 10.3389/fpubh.2024.1429006

**Published:** 2024-10-01

**Authors:** Yongqiang Wang, Yuzhuo Liu, Yulin Chai, Kexuan Chen, Shilan Yang, Xiaochen Feng, Wei Li, Yuqing Mi

**Affiliations:** ^1^School of Management, Shandong Second Medical University, Weifang, Shandong, China; ^2^School of Public Health, Shandong Second Medical University, Weifang, Shandong, China

**Keywords:** health investment, economic development, coupling coordination degree, fuzzy-set qualitative comparative analysis, configuration

## Abstract

**Background:**

System coordination is an effective way to achieve high-quality development, and the debate on the interaction between health investment and economic development is still ongoing. To strengthen previous research and offer feasible advice and references for relevant stakeholders, we provide empirical evidence for exploring intersystem coordination and enhancement pathways using data from China.

**Methods:**

Based on the data published by the National Bureau of Statistics of China, the current status of the interaction and coordination between health investment and economic development in China was measured by calculating the comprehensive evaluation index, relative development degree, and coupling coordination degree. Subsequently, a fuzzy-set qualitative comparative analysis method was introduced to explore pathways for enhancing system interaction and coordination.

**Results:**

There are obvious inter-provincial and regional differences between health investment and economic development in China. Provinces in the west and north are lagging in economic development, while provinces in the east and south are lagging in health investment. There is a clear synergy between health investment and economic development, and there is still much room for improving the degree of coupling coordination between systems. The five conditional configurations derived from the fuzzy-set qualitative comparative analysis yield three pathways for enhancing system coordination: a health expenditure-driven path, an economic development-driven path, and a balanced health investment and economic development-driven path.

**Conclusion:**

Health expenditure is sufficient for high coordination, and the level and equity of investment in health expenditure should be improved. The gross regional product is a necessary and sufficient condition for high coordination, and consideration must be given to strengthening the regional economic support capacity. Health investment and economic development can drive the coordinated development of the system in a balanced way. This enlightens us to give full play to the positive synergy between health investment and economic development based on promoting the benign interaction of subsystems.

## 1 Introduction

Health investment and economic development share a long-standing, dynamic, and interactive relationship (1–4). Health investment directly enhances health levels, while economic development strengthens the capacity of residents to maintain health and improves the accessibility of health services (5, 6). The interplay between these two factors injects a powerful impetus into high-quality development. Therefore, studying the relationship between health investment and economic development has become an essential subject for exploring high-quality development. For this reason, it is necessary for us to systematically review the mechanism of interaction between health investment and economic development. Existing studies have shown that economic development has a positive impact on public health and that this impact has a lagged effect (7–9). Economic development promotes the increase of social and personal investment in health by providing primary material and medical conditions needed for health, thus significantly reducing mortality and prolonging life expectancy (10). Studies have also shown that this effect diminishes once a certain level of economic development is reached (11). Concerning the influence of health investments on economic development, some scholars argue that health investment is an investment in sustainable development. Schultz's human capital theory posited that health investment, as a crucial input of human capital, was an endogenous driver of economic development (12, 13). Studies suggest that health investment, as an implicit factor in economic production, directly fosters economic development (14, 15). Furthermore, with the deepening of research, some scholars believe that health investment indirectly stimulates economic development through intermediary factors such as labor supply, labor productivity, and savings rate (16). From the perspective of health industrialization, successful health investment has stimulated the development of health-related industries, which have become a strong driving force for economic development (17). Based on the literature review above, we can conclude that there is a unique interactive relationship between health investment and economic development.

In the research on the interaction between health investment and economic development, some scholars have confirmed the bidirectional relationship between them. Although health investment and economic development goals differ, they have inherent similarities. Both aim to achieve their respective goals through rational allocation and utilization of human, financial, and material resources. This similarity in internal operation requires subsystems to strengthen coordination in interaction. The COVID-19 epidemic tests the coordination between health investment and economic development. Studies have shown that countries with high levels of coordination between health investment and economic development suffer less during epidemics, and these countries appear more calm and orderly when facing public health crises (18, 19). Therefore, discussing the coordination between subsystems for optimizing health investment and promoting economic development is equally significant. Existing research indicates that studies on system coordination mainly focus on economic and environmental systems. Hou et al. (20) studied the coupling coordination relationship among the economy, ecological environment, and health in China from the perspective of green production. Zhang and Zhao (21) discussed the coordination among green finance, digital economy, and ecological environment in China. These studies aim to explore the coordinated development of various systems. In addition, some studies have also investigated the coordination issues between the healthcare system and the economic system.

For the coupling and coordination relationship between health investment and economic development, scholars mainly discuss the possible logic from the perspective of allocating health and economic resources (22, 23). Initially, researchers used the coupling degree and coordination degree to measure the coupling and coordination relationship between health investment and economic development, which achieved intuitive results. As the research progresses, the researchers clearly define the stages, types, and levels of coordination between health investment and economic development by constructing a coupling coordination model (24–26). These quantitative definitions are based on the actual level of resource allocation, which provides a convincing explanation for system coordination (27). This also provides a methodological foundation for our research. However, we have noticed that current research frequently falls into the passive description of the coupling and coordination relationship between health investment and economic development without actively exploring the path to promote system coupling and coordination under complex conditions. This provides a clear direction for our research.

In summary, examining the relationship between health investment and economic development has made us deeply aware of the critical role played by social subsystems and the importance of coordination between subsystems for high-quality development. This insight has enhanced the theoretical and practical dimensions of our research. Our review of the literature reveals that current studies often pursue singular objectives, lacking a comprehensive, multi-goal approach. Such studies fall short of thoroughly evaluating the levels and coordination of system development. They also neglect to investigate the determinants of system coordination or to propose efficacious means for its enhancement. How can we accurately evaluate the level of health investment and economic development? How can the coordination between subsystems be effectively measured? What constitutes the optimal combination of antecedent conditions to foster system coordination? These challenges are of significant importance and merit in-depth exploration. Addressing them necessitates a highly systematic approach, underscoring the need for comprehensive methodologies in future research.

To extend research on the theme of system coordination and to offer feasible strategic advice and references for relevant stakeholders, we have designed a systematic research methodology to study the interplay between health investment and economic development in China. In terms of the empirical approach, we utilized a systematic evaluation model to assess health investment and economic development and further clarify the relative development levels of subsystems. Subsequently, we explored the interaction and coordination among subsystems by constructing a coupling coordination degree model, clearly defining the coordination stages, types, and levels of health investment and economic development in China. In researching pathways to strengthen system coordination, we positioned our methodology on qualitative comparative analysis (QCA), which has shown favorable outcomes in existing studies (28–30). In exploring interrelationships, Hao et al. (31) employed this method to elucidate the complex relationship between social capital and health from the perspectives of sufficiency and necessity. This approach possesses significant methodological advantages in investigating impact pathways. Li et al. (32) used the fuzzy-set qualitative comparative analysis method to examine the configuration pathways of turnover intention among primary healthcare personnel in Liaoning province. Furthermore, this method has led to important conclusions in many studies (33–36). Therefore, our study integrated the comprehensive evaluation method and the fuzzy-set qualitative comparative analysis to further explore the path to improving the coordination between provincial health investment and economic development in China.

### 1.1 Data sources

The data for this study are obtained from the 2018–2023 “China Statistical Yearbook” and “China Health Care Statistical Yearbook.” The National Bureau of Statistics of China compiled and published these data, encompassing various healthcare and economic development indicators. This database is one of the most comprehensive and authoritative sources of data in China. Our study began at the provincial level, where we collected health investment and economic development data from 31 provinces, autonomous regions, and municipalities in China in 2020 (excluding Taiwan, Hong Kong, and Macao).

### 1.2 Methodology

#### 1.2.1 Integrated development level evaluation model

The comprehensive evaluation index (Equation 1) and the relative development degree (Equation 2) effectively reflect the overall level of development of the system. Therefore, our study selected these indicators to assess the current state of health investment and economic development in China. Initially, we employed the entropy weighting method to determine the weight (***j***) of the indicators (37). Subsequently, based on ***j*** and the standardized values (***X'ij***) of the indicators, a linear weighting method was used to calculate the comprehensive evaluation index for health investment and economic development at the provincial level in China (***Uh*** and ***Ue***) (38). Furthermore, we chose the relative development degree (***S***) to measure the relative level of health investment and economic development at the provincial level in China. According to existing studies, ***S***≥1.2 indicates that the level of health investment exceeds the level of economic development, suggesting a relative lag in economic development; 0.8 < ***S*** < 1.2 indicates a dynamic equilibrium between health investment and economic development, with good interaction and mutual promotion between subsystems; and ***S*** ≤ 0.8 suggests that the level of health investment lags behind economic development and that health investment needs to be increased or optimized to better align with and support economic development (39).


(1) $$\begin{array}{l} U=\overset{m}{\underset{j=1}{\sum }}\omega_{j}{X^{\prime }}_{ij} \end{array}$$

(2) $$\begin{array}{l} S=U_{h}/U_{e} \end{array}$$

#### 1.2.2 Coupling coordination degree model

The coupling coordination degree model (CCDM) is established on the theoretical concepts of “coupling” and “coordination” (40, 41). The coupling degree is usually expressed by **C**, as shown in Equation 3 (42). The coordination degree is reflected by the system coordination index (T), as shown in Equation 4, where **α** and **β** denote the contribution coefficients of the subsystems. The CCDM is a system relationship evaluation model established by synthesizing these two concepts, and the main evaluation index is the coupling coordination degree (**D**), ranging from 0 to 1, as shown in Equation 5 (43). The criteria for determining the coupling coordination phases, types, and grades based on **D** values are presented in Table 1.


(3) $$\begin{array}{l} C=2\times \sqrt{\left[\frac{U_{h}\times U_{e}}{\left(U_{h}+U_{e}\right)\left(U_{h}+U_{e}\right)}\right]} \end{array}$$

(4) $$\begin{array}{l} T=\alpha U_{h}+\beta U_{e} \end{array}$$

(5) $$\begin{array}{l} D=\sqrt{C\times T} \end{array}$$

**Table 1:** Criteria for determining the coupling coordination degree.

**Coupling coordination level**	***D* value range**	**Coupling coordination type**	**Coupling coordination grade**
Low-level coordination (antagonistic phase)	0 ≤ *D* < 0.1	Extreme imbalance	I
	0.1 ≤ *D* < 0.2	Serious imbalance	II
	0.2 ≤ *D* < 0.3	Moderate imbalance	III
	0.3 ≤ *D* < 0.4	Slight imbalance	IV
Medium-level coordination (adjustment phase)	0.4 ≤ *D* < 0.5	Borderline imbalance	V
	0.5 ≤ *D* < 0.6	Barely coordinated	VI
High-level coordination (harmonious phase)	0.6 ≤ *D* < 0.7	Primary coordination	VII
	0.7 ≤ *D* < 0.8	Intermediate coordination	VIII
	0.8 ≤ *D* < 0.9	Good coordination	IX
	0.9 ≤ *D* < 1.0	Excellent coordination	X

#### 1.2.3 Fuzzy-set qualitative comparative analysis

Fuzzy-set qualitative comparative analysis (fs QCA) is a widely applied method within the QCA framework, effectively used to explore the joint effect and interaction relationship (44, 45). The methodological core of fs QCA lies in set theory and Boolean operational logic, which posits that conditional variables do not linearly and independently impact outcome variables but exhibit diversity, concurrency, and equifinality (30, 46). Unlike traditional binary classifications, fs QCA's classification of case conditions and outcomes is not limited to broad binary divisions. It is suitable for evaluating set relationships, such as intersections and inclusions, in complex and ambiguous scenarios where binary conversion is challenging. Furthermore, fs QCA is suitable for macro-level studies with small sample data, enabling effective analysis of various combinations of conditional variables affecting specific outcomes (29, 47). Using configuration methods, fs QCA proposes multiple combinations of conditions to handle complex cases with limited data (48). Given the complex impacts of conditional variables related to health investment and economic development on systemic coordination development, we chose the fs QCA method to analyze pathways for enhancing binary subsystem coordination. The steps in fs QCA primarily include (1) variable labeling, (2) variable calibration, (3) condition testing, (4) truth table calculation, (5) configuration analysis, and (6) robustness testing.

### 1.3 Indicator selection

We selected indicators from the dimensions of health investment and economic development to investigate the comprehensive development level, the status of system coordination, and pathways for enhancing system coordination. In light of the systematic, hierarchical, and obtainable nature of the indicators, we selected appropriate indicators to assess the status of provincial health investment from the perspectives of human investment, material investment, and financial investment, as well as individual investment and government investment, including the number of health technical personnel, per capita health expenditure, per capita healthcare consumption expenditure, and the number of hospital beds. Additionally, indicators such as fixed asset investment, gross regional product, and per capita consumption expenditure were selected to measure economic development from investment, production, and consumption perspectives. Meanwhile, we set the selected evaluation indicators as conditional variables and used the coupling coordination degree as the outcome variable for the configuration analysis.

## 2 Results

### 2.1 Comprehensive development level

#### 2.1.1 Comprehensive evaluation index

**Figure 1:**
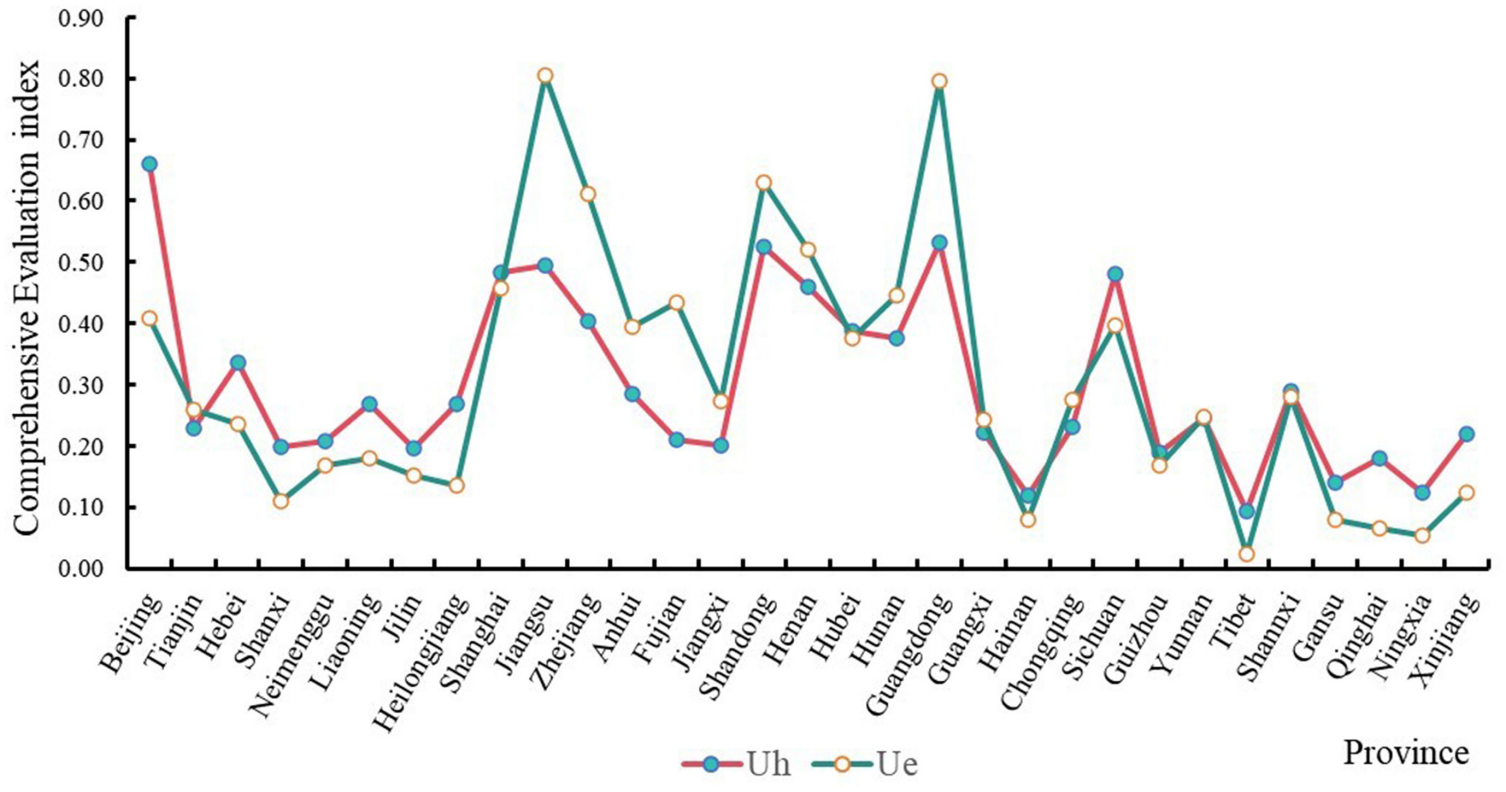
The comprehensive evaluation index of provincial health investment and economic development.

The comprehensive evaluation index of China's health investment and economic development in 2020 is presented in Figure 1 and Table 2. In terms of health investment level, the average value of the comprehensive evaluation index for China's 31 provinces is 0.2989. The comprehensive evaluation indices of Beijing, Guangdong, and Shandong are all above 0.5, indicating a good level of health investment. At the same time, Shanxi, Jilin, Guizhou, Qinghai, Gansu, Ningxia, Hainan, and Tibet have poor health investment levels, with comprehensive evaluation indices < 0.2. In terms of economic development, Jiangsu, Guangdong, Shandong, and Zhejiang exhibit high economic development levels, with comprehensive evaluation indices all above 0.6. In contrast, provinces such as Hainan, Gansu, Qinghai, Ningxia, and Tibet have relatively lower comprehensive economic development indices. Overall, there are significant interprovincial and regional differences in the levels of health investment and economic development in China, with a high correlation between provincial and regional health investment and economic development.

**Table 2:** Comprehensive evaluation index, relative development degree, and coupling coordination degree.

**Province**	***U_*h*_***	***U_*e*_***	***S***	***D***
Beijing	0.655	0.409	1.600	0.720
Tianjin	0.230	0.260	0.885	0.494
Hebei	0.336	0.237	1.414	0.531
Shanxi	0.199	0.110	1.811	0.385
Neimenggu	0.208	0.168	1.234	0.432
Liaoning	0.269	0.180	1.492	0.469
Jilin	0.196	0.151	1.292	0.415
Heilongjiang	0.269	0.136	1.976	0.438
Shanghai	0.484	0.458	1.058	0.686
Jiangsu	0.496	0.805	0.616	0.795
Zhejiang	0.405	0.612	0.662	0.705
Anhui	0.285	0.394	0.723	0.579
Fujian	0.210	0.435	0.482	0.550
Jiangxi	0.202	0.273	0.743	0.485
Shandong	0.525	0.631	0.833	0.759
Henan	0.459	0.521	0.879	0.699
Hubei	0.388	0.377	1.027	0.618
Hunan	0.377	0.447	0.845	0.641
Guangdong	0.533	0.797	0.669	0.807
Guangxi	0.222	0.244	0.909	0.482
Hainan	0.120	0.079	1.520	0.312
Chongqing	0.231	0.275	0.840	0.502
Sichuan	0.480	0.396	1.212	0.660
Guizhou	0.190	0.168	1.130	0.422
Yunnan	0.245	0.248	0.987	0.496
Tibet	0.093	0.024	3.905	0.217
Shannxi	0.290	0.281	1.033	0.534
Gansu	0.140	0.079	1.776	0.324
Qinghai	0.179	0.066	2.731	0.329
Ningxia	0.124	0.055	2.279	0.287
Xinjiang	0.220	0.124	1.849	0.411

#### 2.1.2 Relative development degree

The relative development degrees of health investment and economic development in China in 2020 are detailed in Table 2. Among the 31 provinces, the relative development degree of Xizang, Qinghai, Ningxia, Heilongjiang, Xinjiang, Shanxi, Gansu, Beijing, Hainan, Liaoning, Hebei, Jilin, Neimenggu, and Sichuan exceeds 1.2, indicating a relatively better health investment level compared to economic development. Provinces such as Guizhou, Shanghai, Shaanxi, Hubei, Yunnan, Guangxi, Tianjin, Henan, Hunan, Chongqing, and Shandong have relative development degrees ranging from 0.8 to 1.2, suggesting dynamic coordination and mutual promotion between health investment and economic development. The relative development degrees of Jiangxi, Anhui, Guangdong, Zhejiang, Jiangsu, and Fujian are below 0.8, indicating a relatively slower pace in health investment than economic development. Overall, the level of health investment in China's western and northern provinces is better than the level of economic development. In contrast, health investment tends to be either coordinated or lagging behind economic development in eastern and southern provinces in China.

### 2.2 Coupling coordination relationship

**Figure 2:**
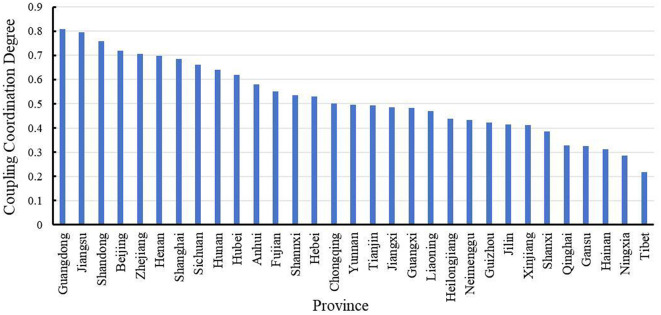
The coupling coordination degree of provincial health investment and economic development.

The degrees of coupling coordination and the types of health investment and economic development in China in 2020 are detailed in Table 2 and Figure 2. Examining the stages, types, and degrees of coupling coordination, provinces such as Guangdong, Jiangsu, Shandong, Beijing, Zhejiang, Henan, Shanghai, Sichuan, Hunan, and Hubei are in the coordination phase. Among these, Guangdong Province shows a high coupling coordination degree of 0.8072, indicating good coordination. Jiangsu, Shandong, Beijing, and Zhejiang are in the intermediate coordination phase, with coupling coordination degrees ranging from 0.7 to 0.8. Henan, Shanghai, Sichuan, Hunan, and Hubei are in the primary coordination phase. Provinces such as Anhui, Fujian, Shaanxi, Hebei, Chongqing, Yunnan, Tianjin, Jiangxi, Guangxi, Liaoning, Heilongjiang, Neimenggu, Guizhou, Jilin, and Xinjiang are in the adjustment phase. In this phase, the coupling coordination degrees of Anhui, Fujian, Shaanxi, Hebei, and Chongqing are between 0.5 and 0.6, indicating a barely coordinated relationship, while the others are on the brink of imbalance. The health investment and economic development of Shanxi, Qinghai, Gansu, Hainan, Ningxia, and Tibet are in the antagonistic phase, with coupling coordination degrees below 0.4. Shanxi, Qinghai, Gansu, and Hainan exhibit slight imbalances, whereas Ningxia and Tibet show moderate imbalances in coupling coordination.

### 2.3 Fuzzy set qualitative comparative analysis

#### 2.3.1 Variable calibration

Before conducting the qualitative comparative analysis, it is essential to calibrate the variables and assign their fuzzy membership values. In our study, we employed the quartile method to determine the quantiles of 75, 50, and 25% of the variables as anchor points of complete unsubordinated, cross-point, and full subordinated, respectively. The calibrated variable data are represented on a scale from [0, 1] (49, 50). To prevent certain cases from being systematically deleted due to difficulties in categorization, the fuzzy membership values of 0.5 were slightly adjusted to 0.501. The fuzzy membership values for the calibrated condition variables and outcome variables are presented in Table 3.

**Table 3:** The fuzzy membership values of calibrated condition variables and outcome variables.

**Province**	**Number of health technicians**	**Per capita health cost**	**Per capita healthcare consumption expenditure**	**Number of beds in medical institutions**	**Investment in fixed assets**	**Gross regional product**	**Consumption expenditure per capita**	***D***
Beijing	0.41	1	1	0.02	0.05	0.86	1	0.98
Tianjin	0	1	1	0	0.15	0.05	1	0.48
Hebei	0.96	0.05	0.15	0.97	0.28	0.86	0.23	0.65
Shanxi	0.35	0.08	0.42	0.21	0.05	0.12	0.01	0.02
Neimenggu	0.05	0.79	0.501	0.05	0.09	0.11	0.73	0.09
Liaoning	0.6	0.18	1	0.71	0.04	0.501	0.87	0.27
Jilin	0.07	0.42	0.95	0.06	0.24	0.03	0.1	0.05
Heilongjiang	0.17	0.91	0.94	0.37	0.13	0.05	0.07	0.1
Shanghai	0.07	1	1	0.04	0.05	0.9	1	0.97
Jiangsu	1	0.95	0.94	1	1	1	1	1
Zhejiang	0.98	0.97	0.8	0.87	0.99	1	1	0.98
Anhui	0.86	0.03	0.05	0.95	0.99	0.9	0.501	0.82
Fujian	0.43	0.29	0.06	0.18	0.94	0.96	1	0.73
Jiangxi	0.501	0.03	0.02	0.57	0.85	0.52	0.21	0.39
Shandong	1	0.39	0.62	1	1	1	0.9	0.99
Henan	1	0.03	0.09	1	1	0.99	0.02	0.98
Hubei	0.88	0.97	0.25	0.95	0.95	0.95	0.6	0.9
Hunan	0.95	0.11	0.96	1	0.99	0.94	0.91	0.93
Guangdong	1	0.91	0.13	1	1	1	1	1
Guangxi	0.77	0.01	0.04	0.62	0.84	0.31	0.03	0.37
Hainan	0	0.77	0.01	0	0.01	0.01	0.53	0
Chongqing	0.15	0.501	0.99	0.27	0.62	0.49	0.95	0.52
Guizhou	0.99	0.47	0.59	1	0.95	0.98	0.73	0.95
Guizhou	0.51	0.02	0	0.52	0.501	0.13	0	0.06
Yunnan	0.76	0.04	0.05	0.76	0.8	0.46	0.05	0.501
Tibet	0	0.95	0	0	0.01	0	0	0
Shannxi	0.75	0.7	0.98	0.501	0.9	0.54	0.11	0.67
Gansu	0.02	0.04	0.05	0.06	0.03	0.01	0.02	0
Qinghai	0	0.99	0.86	0	0.01	0	0.3	0
Ningxia	0	0.77	0.58	0	0.01	0	0.12	0
Xinjiang	0.03	0.96	0.08	0.08	0.1	0.05	0.03	0.04

#### 2.3.2 Analysis of necessary conditions

The necessity of a single condition must be analyzed to judge whether a specific condition is necessary, which can be evaluated by the consistency of the test results (28). Generally speaking, when the consistency of the antecedent condition exceeds 0.900, it indicates that the condition is necessary. Otherwise, it is not considered a necessary condition (44, 51). As illustrated in Table 4, the gross regional product was identified as a necessary condition for the coordination development of health investment and economy. However, the consistency thresholds for the other variables are below 0.900, which suggests that the interaction effect of the variables should be further considered to explore the pathways to improving the coordination of health investment and economic development from the configuration perspective.

**Table 4:** Analysis of necessary conditions for high systemic coordination.

**Condition**	**Consistency**	**Coverage**
Number of health technicians	0.825,788	0.835,528
~Number of health technicians	0.337,413	0.331,025
Per capita health cost	0.601,062	0.568,306
~Per capita health cost	0.521,987	0.549,458
Per capita healthcare consumption expenditure	0.577,035	0.589,637
~Per capita healthcare consumption expenditure	0.481,251	0.467,682
Number of beds in medical institutions	0.774,043	0.809,701
~Number of beds in medical institutions	0.392,397	0.373,114
Investment in fixed asset	0.819,960	0.813,114
~Investment in fixed asset	0.313,451	0.313,695
Gross regional product	0.938,411	0.921,697
~Gross regional product	0.259,115	0.261,863
Consumption expenditure per capita	0.742,892	0.763,664
~ Consumption expenditure per capita	0.353,604	0.341,698

The tilde “~” represents negation.

#### 2.3.3 Configuration analysis

First, the consistency threshold was set to 0.821, the probability margin index consistency threshold was set to 0.700, and the case threshold was set to 1 to construct the truth table (Table 5) (32). Second, since the consistency of gross regional product is >0.900, it was set as necessary (PRESENT). The configuration analysis yielded three solutions in sequence: the complex, parsimonious, and intermediate solutions. Generally, if the antecedent condition appears in both the parsimonious and intermediate solutions, it indicates that the condition is core, and if the antecedent condition appeared only in the intermediate solution, the condition is a fringe condition. According to the existing research, the intermediate solution retains the necessary conditions and simplifies the model, which is a good representation (52). Therefore, the intermediate solution was selected for the configuration analysis. The results of the configuration analysis (Table 6) show that the consistency of the five conditional configurations is >0.900. The consistency and coverage of the overall solutions are 0.977 (>0.800) and 0.818 (>0.500), respectively, which indicates that the five conditional configurations are sufficient conditions for improving the dynamic coordination of health investment and economic development. They can explain ~81.18% of the cases (53).

**Table 5:** The truth table.

**Configuration**	**Number of health technicians**	**Per capita health cost**	**Per capita healthcare consumption expenditure**	**Number of beds in medical institutions**	**Investment in fixed assets**	**Gross regional product**	**Consumption expenditure per capita**	**Case**	**D**	**Consistency**		
										**Raw consistency**	**PRI consistency**	**SYM consistency**
1	1	1	1	1	1	1	1	2	1	0.997	0.997	0.997
2	1	1	0	1	1	1	1	2	1	0.997	0.996	0.996
3	1	0	1	1	1	1	1	3	1	0.997	0.995	0.995
4	1	0	0	1	1	1	1	1	1	0.996	0.992	0.992
5	0	1	1	0	0	1	1	2	1	0.985	0.973	0.973
6	0	0	0	0	1	1	1	1	1	0.984	0.938	0.938
7	1	1	1	1	1	1	0	1	1	0.976	0.943	0.943
8	1	0	0	1	1	1	0	2	1	0.942	0.873	0.873
9	0	1	1	0	1	0	1	1	0	0.932	0.415	0.415
10	1	0	0	1	0	1	0	1	0	0.860	0.545	0.625
11	1	0	1	1	0	1	1	1	0	0.822	0	0
12	1	0	0	1	1	0	0	3	0	0.686	0.002	0.002
13	0	1	1	0	0	0	1	2	0	0.588	0.129	0.167
14	0	0	1	0	0	0	0	1	0	0.357	0	0
15	0	1	0	0	0	0	1	1	0	0.283	0	0
16	0	0	0	0	0	0	0	2	0	0.266	0	0
17	0	1	1	0	0	0	0	3	0	0.228	0	0
18	0	1	0	0	0	0	0	2	0	0.133	0	0

**Table 6:** Configurations for achieving high systemic coordination.

**Condition**	**H1**	**H2**	**H3**	**H4**	**H5**
Number of health technicians	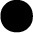	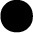	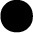	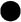	⊗
Per capita health cost		⊗	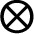	⊗	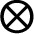
Per capita healthcare consumption expenditure		⊗	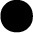	⊗	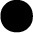
Number of beds in medical institutions	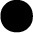	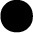	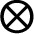	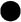	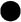
Investment in fixed assets	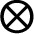	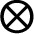	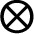	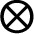	⊗
Gross regional product	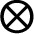	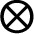	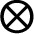	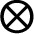	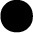
Consumption expenditure per capita	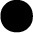			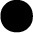	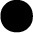
Raw coverage	0.503	0.301	0.276	0.080	0.171
Unique coverage	0.189	0.141	0.025	0.025	0.120
Consistency	0.999	0.947	0.991	0.984	0.985
Solution consistency	0.977				
Solution coverage	0.818				

“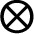,” presence as a core condition; “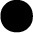,” presence as a peripheral condition; “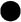,” absent as a core condition; “⊗,” absent as a peripheral condition; blank cells represent ambiguous conditions.

#### 2.3.4 Robustness analyses

We tested the robustness of the results of the configuration analysis by adjusting the consistency threshold and modifying the calibration method (46). Initially, we raised the consistency threshold from 0.821 to 0.85, and the core conditions of the configurations did not change, indicating that the adjustment of the consistency threshold did not substantially alter the study results. Subsequently, without changing the frequency and consistency threshold, we adjusted the calibration anchors for full membership and full non-membership to the 85th percentile and the 15th percentile, respectively. The resulting configurations were broadly consistent with the existing configurations. We then adjusted the cross-calibration anchor to the 45th percentile. After this adjustment, the overall solution coverage experienced slight changes, but the resulting configuration conditions were similar to those before the adjustment. Therefore, the results of the configuration analysis in this study are considered robust.

## 3 Discussion

This study explores possible pathways to improve system coordination by evaluating the level of health investment and economic development, as well as the level of system coordination and interaction in China's provincial areas. The comprehensive evaluation results indicate that the health investment level in the western and northern provinces surpasses economic development. In contrast, the provinces in the eastern and southern regions are predominantly experiencing synergistic or lagging health investment. From the perspective of the current status of system coordination, in 2020, the proportion of provinces in China where health investment and economic development are in the phase of harmonious phase, adjustment phase, and antagonistic phase is 10:15:6. The proportion of provinces with types of good coordination, intermediate coordination, primary coordination, little coordination, borderline imbalance, slight imbalance and moderate imbalance is 1:4:5:5:10:4:2. There is still significant room for improvement in the overall coupling coordination relationship. In addition, based on the five conditional configurations derived from the configuration analysis, we summarize three pathways to enhance the dynamic coordination between health investment and economic development.

Specifically, path 1 is the health expenditure-driven pathway. This path is primarily reflected in H5, where health expenditure is a core condition. This indicates that ensuring government and personal health expenditure can effectively promote coordinated development, particularly when economic development slows down. Stable health expenditure can further ensure investment in health professionals and health infrastructure. Therefore, investment in health expenditure is the key to this path. Beijing and Shanghai are typical cases. Existing research has demonstrated the significant positive impact of health expenditure on health outcomes, with a delayed and long-term sustained effect (10). Hence, the government and individuals should increase health financing and the proportion of health payments. Given the significant interprovincial and regional disparities in health investment and economic development in China, government health investments are needed to reallocate health resources across regions and provinces (54). With the support of health expenditure, it will become more possible to promote the flow of human and material resources, strengthen the unified national health market, and improve the fairness of health investment in terms of population and geography (55, 56). In addition, health investment should aim to improve residents' health and consider the development goals of health undertakings (57). Different investment forms, such as personal nutrition, health care, sports and leisure, and environmental investment, can jointly improve coordinated development.

Path 2 is the economic development-driven pathway, as manifested in H2 and H4. Gross regional product and fixed asset investment are core conditions in this pathway. At the same time, per capita health expenditure and per capita healthcare consumption expenditure are identified as missing marginal conditions. Additionally, the presence of health professionals and the number of hospital beds in H4 are highlighted as core conditions. This path indicates that when a region has a high level of economic development, high-quality system coordination can be achieved even if the effect of health investment is insignificant. Anhui, Fujian, Jiangxi, and Henan are typical cases of this pathway. This is consistent with the research findings of many scholars, who believe that regional economic development is a powerful support for the coordinated development of the system (6, 14). To some extent, gross regional product, as a measure of economic development, influences regional healthcare, while fixed asset investment and residents' consumption capacity play a driving role in coordinated development. Guided by the new development concept, clarifying the key points and focal points of regional economic development is crucial for achieving high-quality development (1). Specifically, it includes the following measures: firstly, increasing labor remuneration and residents' income; secondly, creating a new model of economic development driven by the digital economy; thirdly, narrowing the gap in sharing digital infrastructure; fourthly, adhering to a comprehensive learning system driven by good education; fifthly, enhancing macroeconomic governance capabilities and promoting the modernization of the governance system construction; and sixthly, reinforcing the preemptive position of industrial safety policies (58, 59).

Path 3, as delineated by H1 and H3, represents a balanced-driven pathway of health investment and economic development. In H3, per capita health expenditure, the number of hospital beds, fixed asset investment, and gross regional product are identified as the core conditions. In H1, fixed asset investment and gross regional product are core conditions. This path indicates that strengthening dynamic coordination in the system starts with promoting a balanced development of health investment and regional economy, focusing on building a two-way interaction mechanism and forming a synergistic effect of 1+1>2 (3, 60). The representative provinces of this path include Jiangsu, Zhejiang, Shandong, Hubei, Hunan, Guangdong, and Sichuan. The synergistic effect between health investment and economic development manifests their systemic functionality, with their benign interaction characterized by an orderly and stable scale and structure (61). Empirical studies have shown that the coordination of health investment and economic development has a positive growth effect on the marginal health benefits, driving the development of the health industry. Path 3 elucidates this interactive relationship. To fully harness the positive synergistic effects of the system, the following measures should be implemented: firstly, establishing an all-element open governance system involving health and economic sectors, guided by new development concepts and grounded in networked collaboration; secondly, using health and economic governance as levers to promote the transformation toward a high-quality and health-friendly development model, fostering health economic industry development centered on people's health, integrating health into all policies, comprehensively intervening against health risk factors, creating a healthy environment, and promoting healthy living; finally, in response to regional and inter-provincial differences in coordinated development, we should fully utilize the demonstration effect and spatial spillover effect of high coupling coordination regions to promote efficient interaction between health investment and economic development (55, 56).

Inevitably, our study has certain limitations. Firstly, our study selected a limited number of representative indicators to support our research. However, due to the complexity and multifaceted nature of health investment and economic development, these indicators could not reflect the full range of aspects. Therefore, conducting empirical research that incorporates a broader range of indicators and cases will be an essential task. Secondly, we utilized relatively conventional research tools, which were still insufficient to explain this research topic fully. There remains a need to explore more innovative analytical techniques and methodologies to further investigate the complexities and nuances of the interaction between health investment and economic development. This exploration relies on the establishment of a more systematic theoretical framework and the analysis of influencing mechanisms. Finally, the data used in this study were drawn exclusively from Chinese provinces in 2020, and further theoretical research and methodological applications on this topic should focus on examining the dynamic changes in coordination from a time-series perspective. Nonetheless, the study of China's health investment and economic development level, coordination, and the pathways for improving coordination expands the empirical research exploring the coordinated development aspect of Chinese society. It can provide valuable suggestions for the coordinated development of Chinese society.

## Data Availability

The original contributions presented in the study are included in the article/Supplementary material, further inquiries can be directed to the corresponding authors.
