# Supplementary material for: Synergistic pathways for health investment and economic development in China: a fuzzy-set qualitative comparative analysis
Source: Front Public Health. 2024 Oct 1;12:1429006. doi: 10.3389/fpubh.2024.1429006 (PMC11473372; doi:10.3389/fpubh.2024.1429006)
Supplement: Supplementary file 1 [file Data_Sheet_1.ZIP › Supplementary material/FSQCA.docx]

### Calibration


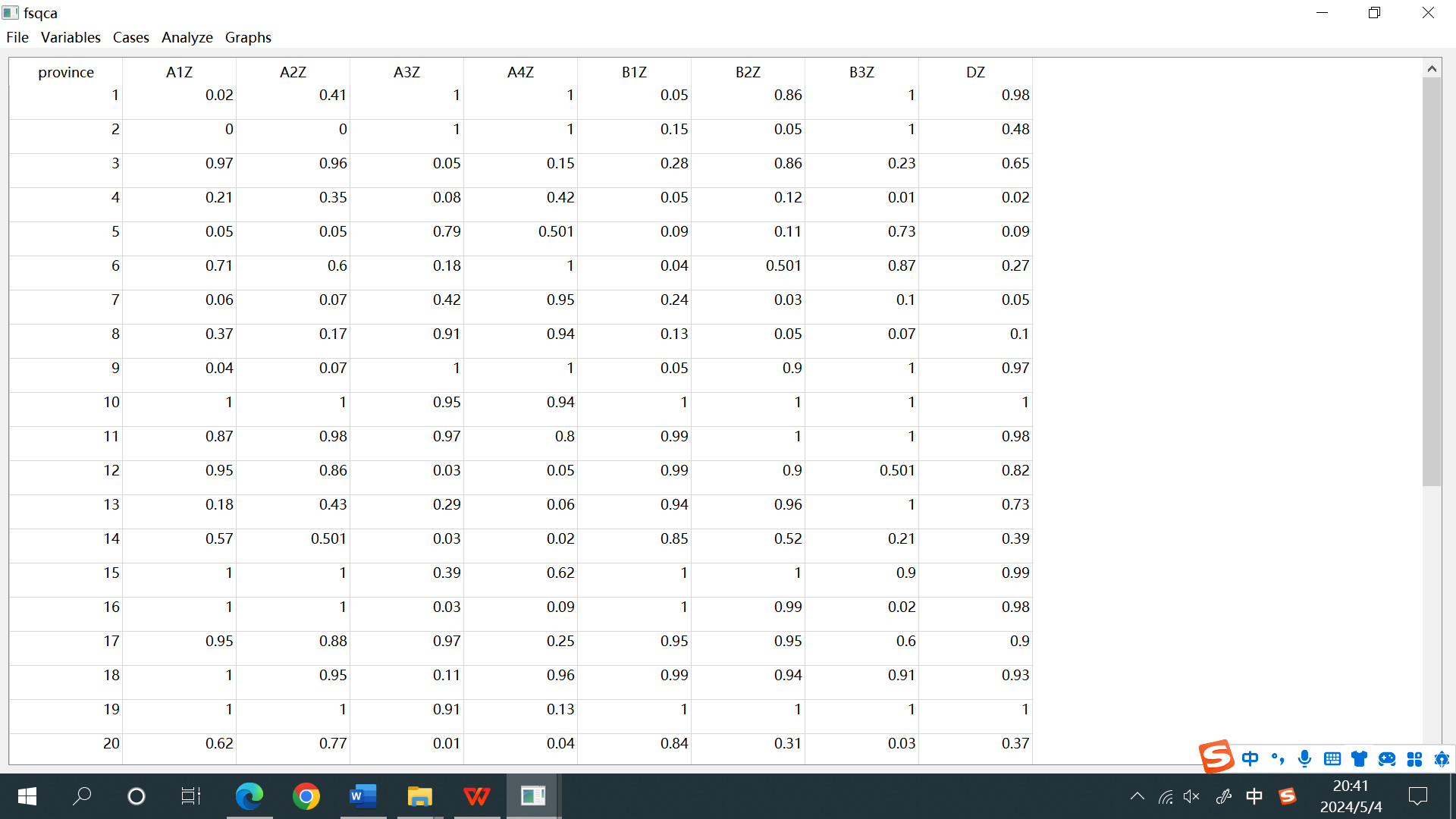


### Necessity analysis


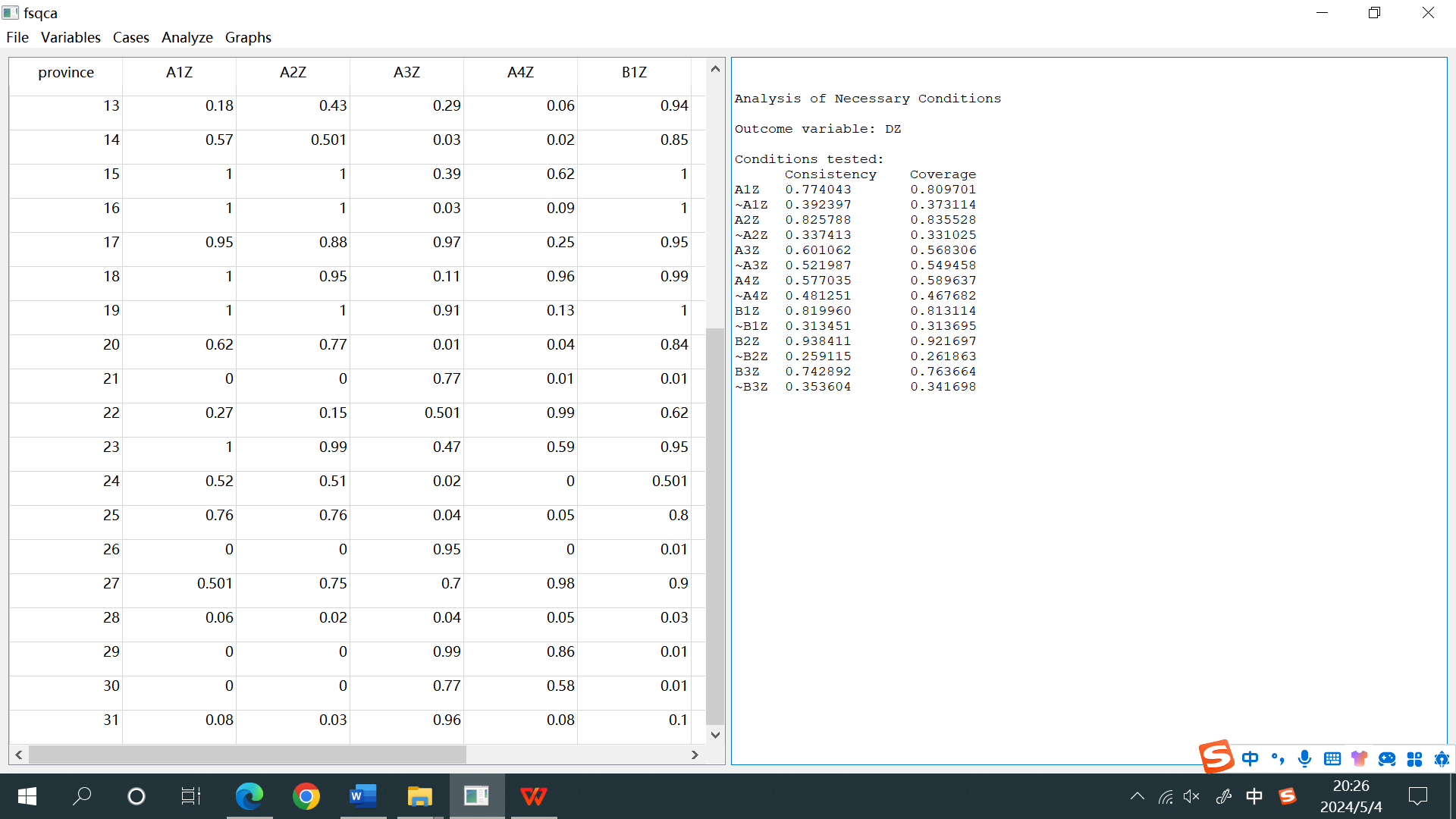


### Sufficiency analysis


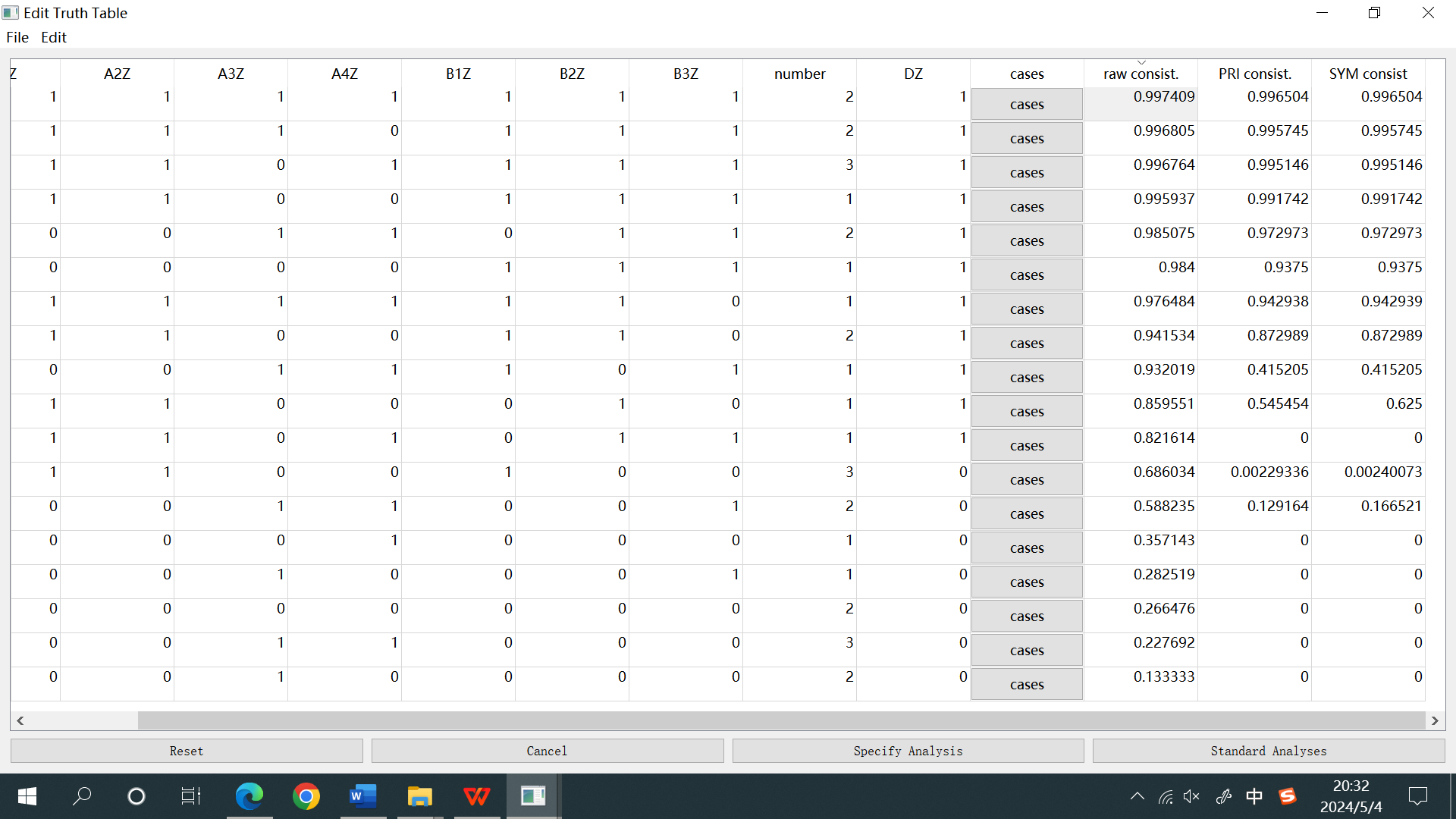


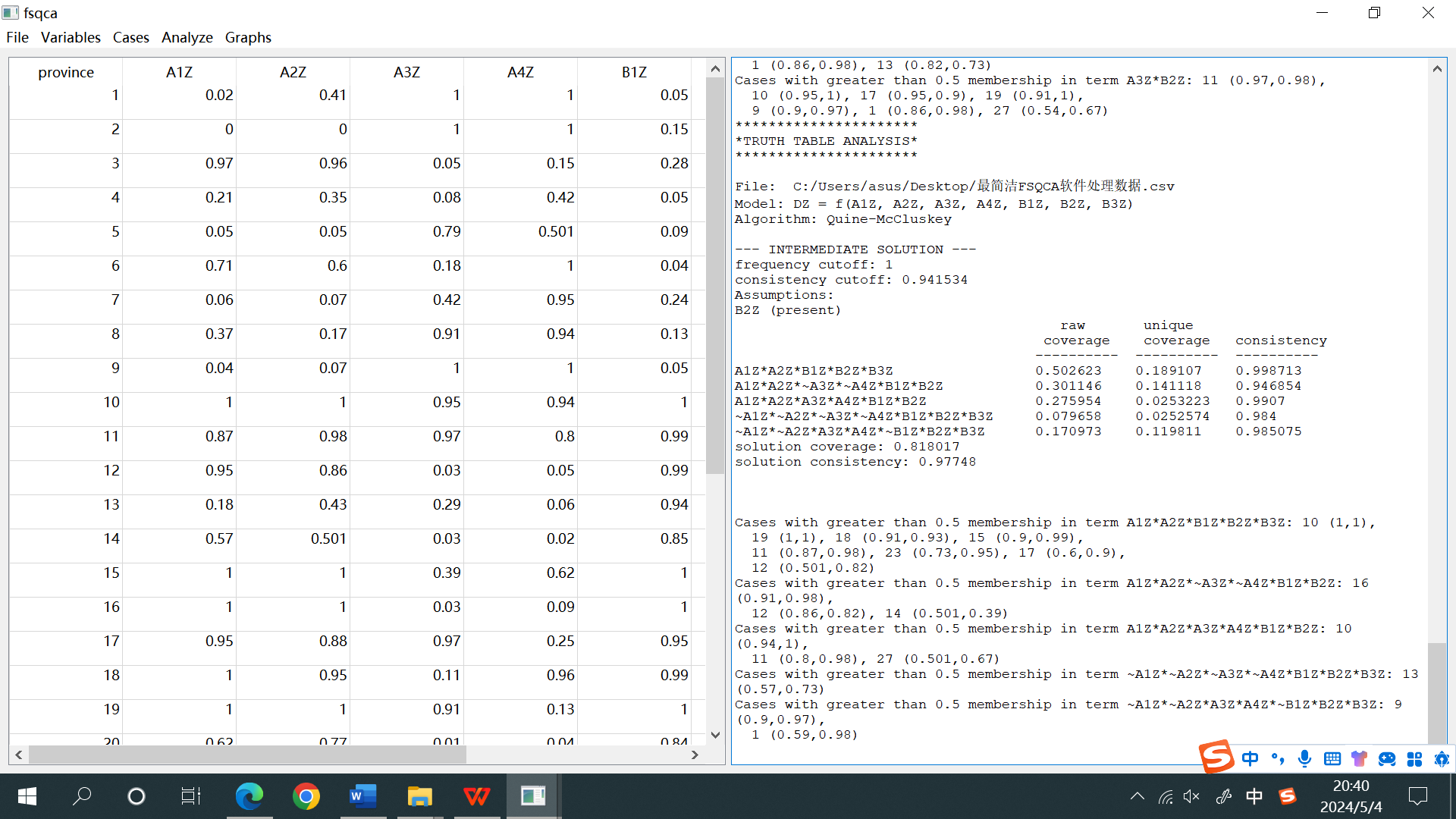


Result:

File: C:/Users/asus/Desktop/最简洁FSQCA软件处理数据.csv

Model: DZ = f(A1Z, A2Z, A3Z, A4Z, B1Z, B2Z, B3Z)

Algorithm: Quine-McCluskey

--- COMPLEX SOLUTION ---

frequency cutoff: 1

consistency cutoff: 0.941534

raw unique

coverage coverage consistency

---------- ---------- ----------

A1Z*A2Z*B1Z*B2Z*B3Z 0.502623 0.189107 0.998713

A1Z*A2Z*~A3Z*~A4Z*B1Z*B2Z 0.301146 0.141118 0.946854

A1Z*A2Z*A3Z*A4Z*B1Z*B2Z 0.275954 0.0253223 0.9907

~A1Z*~A2Z*~A3Z*~A4Z*B1Z*B2Z*B3Z 0.079658 0.0252574 0.984

~A1Z*~A2Z*A3Z*A4Z*~B1Z*B2Z*B3Z 0.170973 0.119811 0.985075

solution coverage: 0.818017

solution consistency: 0.97748

Cases with greater than 0.5 membership in term A1Z*A2Z*B1Z*B2Z*B3Z: 10 (1,1),

19 (1,1), 18 (0.91,0.93), 15 (0.9,0.99),

11 (0.87,0.98), 23 (0.73,0.95), 17 (0.6,0.9),

12 (0.501,0.82)

Cases with greater than 0.5 membership in term A1Z*A2Z*~A3Z*~A4Z*B1Z*B2Z: 16 (0.91,0.98),

12 (0.86,0.82), 14 (0.501,0.39)

Cases with greater than 0.5 membership in term A1Z*A2Z*A3Z*A4Z*B1Z*B2Z: 10 (0.94,1),

11 (0.8,0.98), 27 (0.501,0.67)

Cases with greater than 0.5 membership in term ~A1Z*~A2Z*~A3Z*~A4Z*B1Z*B2Z*B3Z: 13 (0.57,0.73)

Cases with greater than 0.5 membership in term ~A1Z*~A2Z*A3Z*A4Z*~B1Z*B2Z*B3Z: 9 (0.9,0.97),

1 (0.59,0.98)

**********************

*TRUTH TABLE ANALYSIS*

**********************

File: C:/Users/asus/Desktop/最简洁FSQCA软件处理数据.csv

Model: DZ = f(A1Z, A2Z, A3Z, A4Z, B1Z, B2Z, B3Z)

Algorithm: Quine-McCluskey

--- PARSIMONIOUS SOLUTION ---

frequency cutoff: 1

consistency cutoff: 0.941534

raw unique

coverage coverage consistency

---------- ---------- ----------

B1Z*B2Z 0.792047 0.26747 0.950272

~A2Z*B2Z 0.301146 0 0.910158

~A1Z*B2Z 0.34253 0 0.934617

A3Z*B2Z 0.547244 0 0.981417

solution coverage: 0.914449

solution consistency: 0.94259

Cases with greater than 0.5 membership in term B1Z*B2Z: 10 (1,1),

15 (1,0.99), 19 (1,1), 11 (0.99,0.98),

16 (0.99,0.98), 17 (0.95,0.9), 23 (0.95,0.95),

13 (0.94,0.73), 18 (0.94,0.93), 12 (0.9,0.82),

27 (0.54,0.67), 14 (0.52,0.39)

Cases with greater than 0.5 membership in term ~A2Z*B2Z: 9 (0.9,0.97),

1 (0.59,0.98), 13 (0.57,0.73)

Cases with greater than 0.5 membership in term ~A1Z*B2Z: 9 (0.9,0.97),

1 (0.86,0.98), 13 (0.82,0.73)

Cases with greater than 0.5 membership in term A3Z*B2Z: 11 (0.97,0.98),

10 (0.95,1), 17 (0.95,0.9), 19 (0.91,1),

9 (0.9,0.97), 1 (0.86,0.98), 27 (0.54,0.67)

**********************

*TRUTH TABLE ANALYSIS*

**********************

File: C:/Users/asus/Desktop/最简洁FSQCA软件处理数据.csv

Model: DZ = f(A1Z, A2Z, A3Z, A4Z, B1Z, B2Z, B3Z)

Algorithm: Quine-McCluskey

--- INTERMEDIATE SOLUTION ---

frequency cutoff: 1

consistency cutoff: 0.941534

Assumptions:

B2Z (present)

raw unique

coverage coverage consistency

---------- ---------- ----------

A1Z*A2Z*B1Z*B2Z*B3Z 0.502623 0.189107 0.998713

A1Z*A2Z*~A3Z*~A4Z*B1Z*B2Z 0.301146 0.141118 0.946854

A1Z*A2Z*A3Z*A4Z*B1Z*B2Z 0.275954 0.0253223 0.9907

~A1Z*~A2Z*~A3Z*~A4Z*B1Z*B2Z*B3Z 0.079658 0.0252574 0.984

~A1Z*~A2Z*A3Z*A4Z*~B1Z*B2Z*B3Z 0.170973 0.119811 0.985075

solution coverage: 0.818017

solution consistency: 0.97748

Cases with greater than 0.5 membership in term A1Z*A2Z*B1Z*B2Z*B3Z: 10 (1,1),

19 (1,1), 18 (0.91,0.93), 15 (0.9,0.99),

11 (0.87,0.98), 23 (0.73,0.95), 17 (0.6,0.9),

12 (0.501,0.82)

Cases with greater than 0.5 membership in term A1Z*A2Z*~A3Z*~A4Z*B1Z*B2Z: 16 (0.91,0.98),

12 (0.86,0.82), 14 (0.501,0.39)

Cases with greater than 0.5 membership in term A1Z*A2Z*A3Z*A4Z*B1Z*B2Z: 10 (0.94,1),

11 (0.8,0.98), 27 (0.501,0.67)

Cases with greater than 0.5 membership in term ~A1Z*~A2Z*~A3Z*~A4Z*B1Z*B2Z*B3Z: 13 (0.57,0.73)

Cases with greater than 0.5 membership in term ~A1Z*~A2Z*A3Z*A4Z*~B1Z*B2Z*B3Z: 9 (0.9,0.97),

1 (0.59,0.98)
