# Supplementary figures and images for: Synergistic pathways for health investment and economic development in China: a fuzzy-set qualitative comparative analysis
Source: Front Public Health. 2024 Oct 1;12:1429006. doi: 10.3389/fpubh.2024.1429006 (PMC11473372; doi:10.3389/fpubh.2024.1429006)

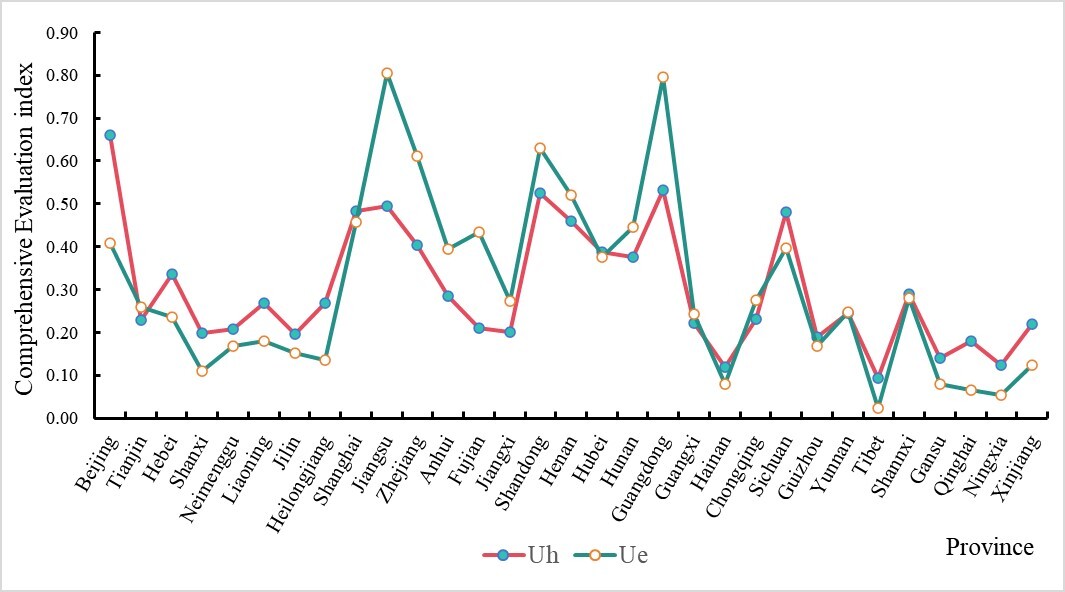

Supplement: Supplementary file 1 [file Data_Sheet_1.ZIP › Supplementary material/Figure 1.jpg]

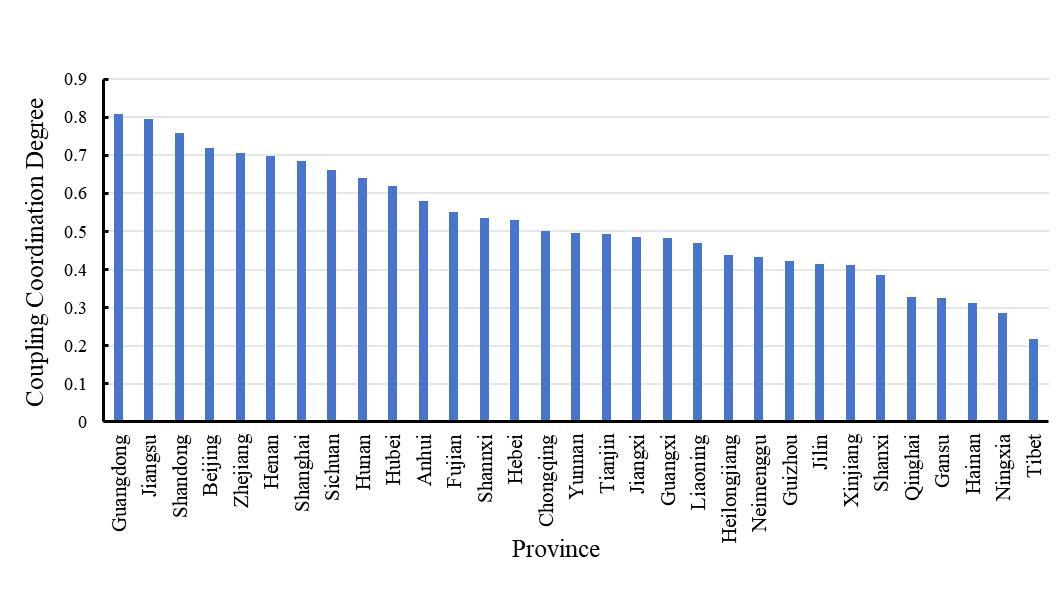

Supplement: Supplementary file 1 [file Data_Sheet_1.ZIP › Supplementary material/Figure 2.jpg]
